# Daily Intake of Grape Powder Prevents the Progression of Kidney Disease in Obese Type 2 Diabetic ZSF1 Rats

**DOI:** 10.3390/nu9040345

**Published:** 2017-03-31

**Authors:** Salwa M. K. Almomen, Qiunong Guan, Peihe Liang, Kaidi Yang, Ahmad M. Sidiqi, Adeera Levin, Caigan Du

**Affiliations:** 1 Department of Urologic Sciences, University of British Columbia, Vancouver, BC V6H3Z6, Canada; sa.momen@windowslive.com (S.M.K.A.); qiunong@hotmail.com (Q.G.); lph1972@163.com (P.L.); kaidiyang1994@gmail.com (K.Y.); ahmad.sidiqi@alumni.ubc.ca (A.M.S.); 2 Immunity and Infection Research Centre, Vancouver Coastal Health Research Institute, Vancouver, BC V6H3Z6, Canada; 3 Department of Urology, The Second Affiliated Hospital, Chongqing Medical University, Chongqing 400010, China; 4 Division of Nephrology, Department of Medicine, University of British Columbia, Vancouver, BC V6Z1Y6, Canada; alevin@providencehealth.bc.ca

**Keywords:** metabolic syndrome, chronic kidney disease, grape powder, antioxidants, natural products, dietary supplements

## Abstract

Individuals living with metabolic syndrome (MetS) such as diabetes and obesity are at high risk for developing chronic kidney disease (CKD). This study investigated the beneficial effect of whole grape powder (WGP) diet on MetS-associated CKD. Obese diabetic ZSF1 rats, a kidney disease model with MetS, were fed WGP (5%, *w*/*w*) diet for six months. Kidney disease was determined using blood and urine chemical analyses, and histology. When compared to Vehicle controls, WGP intake did not change the rat bodyweight, but lowered their kidney, liver and spleen weight, which were in parallel with the lower serum glucose and the higher albumin or albumin/globin ratio. More importantly, WGP intake improved the renal function as urination and proteinuria decreased, or it prevented kidney tissue damage in these diabetic rats. The renal protection of WGP diet was associated with up-regulation of antioxidants (*Dhcr24*, *Gstk1*, *Prdx2*, *Sod2*, *Gpx1 and Gpx4*) and downregulation of *Txnip* (for ROS production) in the kidneys. Furthermore, addition of grape extract reduced H_2_O_2_-induced cell death of cultured podocytes. In conclusion, daily intake of WGP reduces the progression of kidney disease in obese diabetic rats, suggesting a protective function of antioxidant-rich grape diet against CKD in the setting of MetS.

## 1. Introduction

Chronic kidney disease (CKD) is multifactorial, and is defined as abnormalities of kidney structure or function that is present for more than three months. While not progressive in all, it can lead to end-stage renal disease (ESRD)—total and permanent kidney failure [1,2]. CKD places a major public health burden on our community as it affects more than 10% of adults aged 20 years or older, or more than 40% in those aged 65 years or older in the United States (source: Centers for Disease Control and Prevention), and 12.5% of adults, representing approximately three million, in Canada [3,4]. Like other chronic diseases, many cases of CKD are not curable, but there are substantial numbers of people who can benefit from prevention or delay of progression [5], suggesting that an effective and feasible preventive strategy is expected to reduce the burden of CKD in our community. However, there are a limited number of these that are proven or used in our daily practice. Recently, there is an increasing interest in diet, the interaction of gut microbiome and CKD progression. Dietary supplements or natural health products is definitely an attractive and feasible strategy for CKD prevention. 

Metabolic syndrome (MetS) is a collective term for several clinical measures, which include insulin resistance, high serum glucose, overweight or obesity, high triglyceride or low high-density lipoprotein cholesterol and hypertension [6]. Literature suggests that MetS may increase the risk of the development of CKD [7,8,9], mainly seen as hypertensive and diabetic nephropathy (DN). While the etiology for MetS-induced CKD remains unknown [9], oxidative stress is theorized to a play a role [8,10,11]. The oxidative stress is induced by the imbalance between the level of reactive oxygen species (ROS, free radicals) and the capacity of antioxidant defenses, which leads to the generation of excessive ROS and consequently tissue damage [11,12]. One of major antioxidant defense mechanisms in our body is exogenous antioxidants, such as flavonoids, vitamins, resveratrol, anthocyanine, curcumin and phenolic acid that come from our food and other dietary sources [11,13,14,15]. There are some data that antioxidant rich berry diet has benefit in preventing or mitigating MetS [15,16], suggesting that a dietary strategy of using antioxidant-rich food may be a novel strategy to employ to reduce the incidence of CKD among individuals who are living with MetS.

Grapes are one of the most widely cultivated and popularly consumed fruits in the world, and contain over 1600 phytonutrients including some major antioxidant flavonoids, such as catechins (catechin and epicatechin), anthocyanins (peonidin, cyaniding, and malvidin), flavonols (quercetin, kaempferol, and isorhamnetin) and resveratrol (source: the California Table Grape Commission report, Fresno, CA, USA). The renoprotective activities of these compounds have been demonstrated in various experimental models. For example, epicatechin, quercetin or resveratrol has been shown to lower the oxidative stress in the kidneys and reduce renal damage in animals with cisplatin nephropathy [17,18,19], and feeding with anthocyanin-rich food reduces diabetes-associated kidney failure in db/db mice [20]. A recent study shows that daily drinking of whole grape powder (WGP) prevents age-related decline of kidney function in rats [21], but its beneficial effect on MetS-associated CKD, a common public health problem in our community [22], has not been investigated. Obese ZSF1 male rats are generated by a cross between a Zucker diabetic fatty (ZDF) female and a spontaneously hypertensive heart failure (SHHF) male rat, which have MetS similar to humans, including hypertension, type 2 diabetes, hyperlipidemia and nephropathy [23,24,25,26,27]. Hence, these rats have been widely used as a disease model for renal failure in the condition of MetS [27,28,29,30]. The objective of this study was to examine the effect of daily feeding with the WGP on the progression of kidney disease in obese ZSF1 rats, as compared to control animals.

## 2. Materials and Methods 

### 2.1. Animals and Cells

Obese ZSF1 male rats (~300 g bodyweight, 8 weeks old) were purchased from Charles River Laboratories International, Inc. (Wilmington, MA, USA). These rats were F1 hybrids from a cross between a female ZDF rat and a male SHHF rat [27], and were maintained in the animal facility of the Jack Bell Research Centre (Vancouver, BC, Canada). All animal experiments were performed in accordance with the Canadian Council on Animal Care guidelines under protocols approved by the Animal Use Subcommittee at the University of British Columbia (Vancouver, BC, Canada). 

Heat-sensitive mouse podocyte (HSMP) cell line was a gift from Dr. Stuart Shankland (University of Washington School of Medicine, Seattle, WA, USA). HSMP cells were maintained and grown in RPMI 1640 culture medium supplemented with 10% fetal bovine serum and 0.2 ng·mL^−1^ of IFN-γ in a CO_2_ incubator at 33 °C as described previously [31]. To induce quiescence and the differentiated phenotype for experiments, HSMP cells were grown at 37 °C in the same medium but in the absence of IFN-γ (growth restrictive conditions) [31].

### 2.2. Whole Grape Powder (WGP)

WGP was provided by the California Table Grape Commission (CTGC), and was a freeze-dried grape product developed for the usage of research purposes only. WGP was composed of seeded and seedless varieties of fresh green, red and black California grapes that were initially frozen, and then ground with food-quality dry ice. The processing and storage of the WGP were done properly for preserving the bioactivities of the compounds found in the grapes. Approximately 90% of the WGP by weight was sugars (glucose and fructose found in a 1:1 ratio), and the remainder consisted of organic acids, phenolic compounds, nitrogenous compounds, aroma compounds, and minerals and pectic substances. Antioxidants, such as resveratrol, flavans (including catechin), flavonols (including quercetin), anthocyanins and many simple phenolics (as per the CTGC report) were also found. This product was stored at −80 °C in moisture-proof containers until it was mixed with regular rat chow for the animal experiments. 

### 2.3. Food Supplements 

According to the CTGC guideline of the usage of WGP as food supplements for animal studies, WGP supplement food (denoted as WGP here) was prepared for this study by mixing regular chow (95%, *w*/*w*) with WGP (5%, *w*/*w*), and its Vehicle control the regular chow (95.5%, *w*/*w*) with 2.25% (*w*/*w*) glucose and 2.25% (*w*/*w*) fructose. Rats were randomly assigned to two groups (WGP and Vehicle), in which they were fed with either WGP or sugar control. The animal experiment was repeated twice with total 15 rats in each group. One rat in WGP group suffered a urinary tract infection diagnosed early during the second month of the feeding experiment, and was consequently excluded from the experiment and data analysis.

### 2.4. Preparation of WGP Extract 

The WGP extract was prepared for both cell culture experiments to determine its antioxidative activity. In brief, WGP (4 g) was dissolved in 200 mL of methanol (anhydrous, 99.8%) and was stirred for 72 h at room temperature, followed by centrifugation at 3000 rpm for 15 min. The resultant supernatant fraction was subsequently dried by lyophilization using a centrifugal evaporator. Around 3.3 g of WGP-methanol extract was produced from 4 g of WGP by this procedure. The stock solution of WGP extract was prepared by dissolving the dried extract pellet in culture medium at the concentration of 5 mg·mL^−1^.

### 2.5. Primary Outcomes from This Study

Our primary outcomes were to measure renal function through assessment of urine volume, proteinuria, urine protein to creatinine ratio (uPCR), and estimated glomerular filtration rate (GFR). Renal injury was also measured via the kidney organ index and histological analysis. 

### 2.6. Urine Collection and Determination of Kidney Function

A 24-h urine sample was collected using metabolic caging, in which the rat had unrestricted access to food and drinking water. The collected urine was immediately centrifuged at 3000 rpm for 15 min to remove any food scraps and fecal matters. After its volume was measured, the sample was stored in aliquots at −20 °C until all the samples were collected for the determination of both protein and creatinine levels at the same time. 

The levels of urine creatinine (UCr) were measured in the Chemistry Laboratory at the Vancouver Coastal Health Regional Laboratory Medicine (Vancouver, BC, Canada) by using the Dimension Vista^®^ System with CRE2 reagent cartridges (Siemens Healthcare Diagnostics Inc., Newark, DE, USA). The levels of urine protein were determined using Bio-Rad protein assay following manufacturer’s instruction (Bio-Rad Laboratories-Canada, Mississauga, ON, Canada). In brief, the protein in the urine was precipitated using 10% trichloroacetic acid, followed by centrifugation at 10,000 rpm for 10 min. The protein pellet was suspended in 3% sodium hydroxide (NaOH) solution, and 1 μL of this protein solution was added to 200 μL of Bio-Rad dye (10 × dilution). The optical density (OD) of urine protein samples, background control and bovine serum albumin (BSA) standards at 595 nm was measured using an ELx808 Ultra Microplate Reader (BioTek, Winooski, VT, USA), and protein concentration (mg·mL^−1^) in the urine sample was calculated based on the BSA standard curve. 

### 2.7. Blood Collection and Chemical Analysis 

Blood samples were collected from the tail vein using a lithium heparin tube after 1, 3 and 6 months of WGP feeding. A comprehensive metabolic panel of 14 blood substances, including alanine aminotransferase (ALT), albumin (ALB), alkaline phosphatase (ALP), amylase (AMY), total calcium (Ca^2+^), creatinine (CRE), globulin (GLOB), glucose (GLU), phosphorus (PHOS), potassium (K^+^), sodium (Na^+^), total bilirubin (TBIL), total protein (TP), and urea nitrogen (BUN), were determined using the VetScan^®^ Comprehensive Diagnostic Profile reagent rotor with the VetScan Chemistry Analyzer VS2 (Abaxis, Inc., Union City, CA, USA) in the Animal Unit Hematology Diagnostic Laboratory at Jack Bell Research Centre (Vancouver, BC, Canada).

### 2.8. Glomerular Filtration Rate (GFR) 

The renal function was estimated using GFR that was calculated based on the creatinine clearance using the following: GFR = (UCr × V)/SCr, where UCr was creatinine level in urine sample, V the volume of the 24-h urine sample, and SCr the creatinine in serum.

### 2.9. Renal Histopathology

The kidney tissues (6 rats/group) were randomly selected. The tissues were fixed in 10% neutral buffered formalin, followed by embedding in paraffin wax. Sections were cut at 4-μm thickness, and were stained with hematoxylin and eosin (HE) or periodic acid-Schiff (PAS). The stained tissue sections were scanned with Leica SCN400 Slide scanner (Leica Microsystens Inc., Concord, ON, Canada), and the pathological parameters of renal disease were determined in two separate sections of each kidney using the Digital Image Hub—A slidepath Software Solution (Leica Microsystems Inc.) in a blinded fashion. 

The number of intratubular protein cast formation or glomerular atrophy in each microscopic view was counted in HE-stained section, and the average number of at least 20 randomly selected views under 40× magnification represented these two parameters in each kidney. 

A semi-quantitative scoring system using a 0 to 4 scale was established to determine the severity of the tubular atrophy (including tubular dilution and/or cellular flatting) in the renal cortex of HE-stained kidney sections. The scale/score was measured based on the percentage of damaged tubules occupying a microscopic view: 1 (0%–24% of the area affected with damaged tubules), 2 (25%–49%), 3 (50%–74%), and 4 (>75%). The average of at least 30 randomly selected views under 200× magnification represented the tubular atrophy in each kidney. 

The mesangial expansion was also determined using a 0 to 4 scale based on the percentage of the area stained strongly with PAS, indicating the mesangial expansion in an affected glomerulus: 1 (0%–24% of the area affected with densely stain, minimal ), 2 (25%–49%, mild), 3 (50%–74%, moderate), and 4 (>75%, severe). A range of 180 to 250 glomeruli were counted and averaged for each kidney. 

### 2.10. PCR Array Analysis of Oxidative Stress

The expression of 84 oxidative stress-associated genes in kidney tissues was quantitatively examined using PCR Arrays kits (Cat. No.: PARN-065Z, SABiosciences—QIAGEN Inc., Valencia, CA, USA) following manufacturer’s instruction. Four samples/rats (one from each rat) were randomly selected from each group. In brief, one piece of kidney cortex was snap-frozen in liquid nitrogen and stored at −80 °C until all the samples were ready for the experiment. The total RNA from the tissues was extracted and purified using the RNeasy Microarray Tissue Mini kit (QIAGEN), and converted to cDNA using RT^2^ First Strand Kit (QIAGEN). The expression of selected genes was amplified by real-time PCR using RT^2^ Profile PCR arrays (QIAGEN). Data were analyzed using Web-based PCR Array Data Analysis Software at the website of the manufacturer (http://www.SABiosciences.com/pcrarraydataanalysis.php).

### 2.11. Flow Cytometric Analysis 

Cell death or viability was quantitatively determined by using fluorescence-activated cell sorter (FACS) analysis following the manufacturer’s protocol (BD Biosciences, Mississauga, ON, Canada). Annexin-V conjugated with phycoerythrin (Annexin-V-PE) stained apoptotic cells, and 7-amino-actinomycin D (7-AAD) positivity indicated late apoptotic and necrotic cells. Briefly, cells were stained with both Annexin-V-PE and 7-AAD for 15 min in the dark, and cell apoptosis and necrosis were detected by using a flow cytometry and further quantified using FlowJo software (Tree Star Inc., Ashland, OR, USA). In a FACS graph, the lower right quadrant—only Annexin V positive indicated early apoptosis; the upper right quadrant—Annexin V/7-AAD double positive showed late apoptosis; and the upper left quadrant—7-AAD positive cells were necrosis. Finally, negative staining (the lower left quadrant—Annexin V/7-AAD double negative) showed the population of viable cells. 

### 2.12. Statistical Analysis 

Data were presented as mean ± standard derivation (SD) of each group. Statistical analysis of difference between groups was performed using GraphPad Prism software (GraphPad, San Diego, CA, USA) by *t*-test or analysis of variance (ANOVA) as indicated in the text. A *p* value of ≤0.05 was considered statistically significant.

## 3. Results

### 3.1. Daily Intake of WGP Is Associated with Lower Organ Index of the Kidney, Liver and Spleen but Does Not Affect Bodyweight in Obese Diabetic Rats

**Figure 1:**
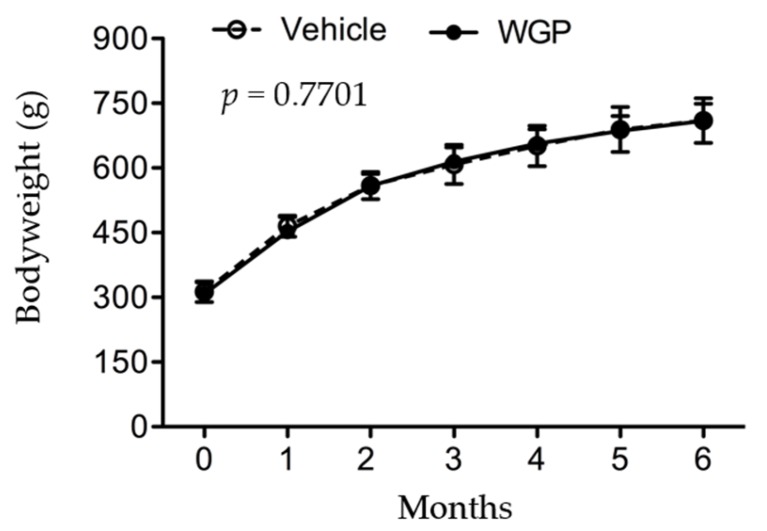
No effect of daily intake of whole grape powder (WGP) on bodyweight gain or obesity in obese diabetic ZSF1 rats.

Both bodyweight and diet consumption were monitored once a week. As shown in Figure 1, the weight gain of rats during six months of WGP feeding was the same as sugar-fed Vehicle control group, indicated by a complete overlap of growth curves between these two groups (WGP vs. Vehicle: *p* = 0.7701, two-way ANOVA). This observation was correlated with their diet consumption, which remained the same at 25 ± 2 g·day^−1^·animal^−1^ between these two groups throughout the experiment. Interestingly, at the end of feeding experiment, significantly lower organ weight index was seen in the kidney (WGP vs. Vehicle: *p* = 0.0127, two-tailed *t*-test), liver (WGP vs. Vehicle: *p* < 0.0001, two-tailed *t*-test) and spleen (WGP vs. Vehicle: *p* = 0.0341, two-tailed *t*-test) of WGP-fed rats compared to Vehicle controls, whereas both the heart and the lung remained the same (Table 1). The data suggested that daily intake of WGP reduced the weight/size of these three organs—the liver, kidney and spleen—as compared to those in control group in these obese diabetic rats.

The bodyweight of rats in both Vehicle (*n* = 15) and WGP (*n* = 14) groups was recorded during a period of six months of dietary supplements. Data were presented as mean ± SD of each group. Vehicle vs. WGP: *p* = 0.7701 (two-way ANOVA).

### 3.2. Daily Intake of WGP Is Associated with Higher Serum Albumin and Lower Blood Glucose in Obese Diabetic Rats 

**Figure 2:**
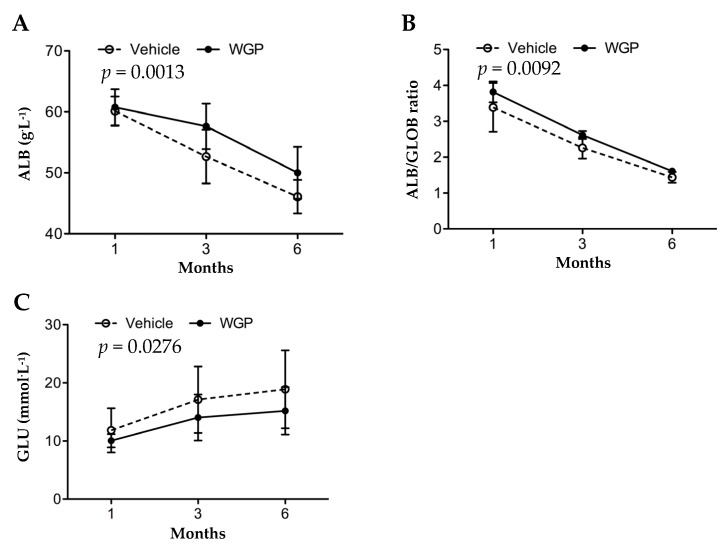
Daily intake of WGP has beneficial effect on the maintenance of blood albumin and the reduction of blood glucose in obese diabetic ZSF1 rats. The blood levels of albumin (ALB), globulin (GLOB) and glucose (GLU) were measured in randomly selected rats in Vehicle (*n* = 8–12) or WGP (*n* = 8–11) group after one, three or six months of dietary supplements. Data were presented as mean ± SD of each group. (**A**) Blood ALB levels. Vehicle vs. WGP: *p* = 0.0013 (two-way ANOVA); (**B**) ALB/GLOB ratio. Vehicle vs. WGP: *p* = 0.0092 (two-way ANOVA); (**C**) Blood GLU levels. Vehicle vs. WGP: *p* = 0.0276 (two-way ANOVA).

The overall metabolism status of these rats was determined by using the changes in a comprehensive metabolic panel of 14 blood substances that represent the basic metabolism (GLU), electrolytes (Na^+^, K^+^, Ca^2+^, and PHOS) and both kidney and liver functions (BUN, CRE, ALB, GLOB. ALB/GLOB ratio, ALP, ALT, AMY, TP and TBIL). Over six months of feeding experiment, the rats in both groups experienced a significant decline in ALB (including ALB/GLOB ratio), ALP, BUN, K^+^ and PHOS, and at the same time an increase in GLU, AMY, TBIL, GLOB, TP, Ca^2+^ and Na^+^ (Table S1). Only CRE and ALT remained unchanged (Table S1). The importance for this examination was that as compared to the Vehicle group, WGP-fed rats had significantly higher levels of serum ALB (*p* = 0.0013, two-way ANOVA) or ALB/GLOB ratio (*p* = 0.0092, two-way ANOVA), and lower levels of blood GLU (*p* = 0.0276, two-way ANOVA) (Table S1 and Figure 2), at the end of the six-month period.

### 3.3. Daily Intake of WGP Significantly Is Associated with Lower Urine Volume and Urine Protein Excretion but Does Not Impact GFR after Six Months in Obese Diabetic Rats 

**Figure 3:**
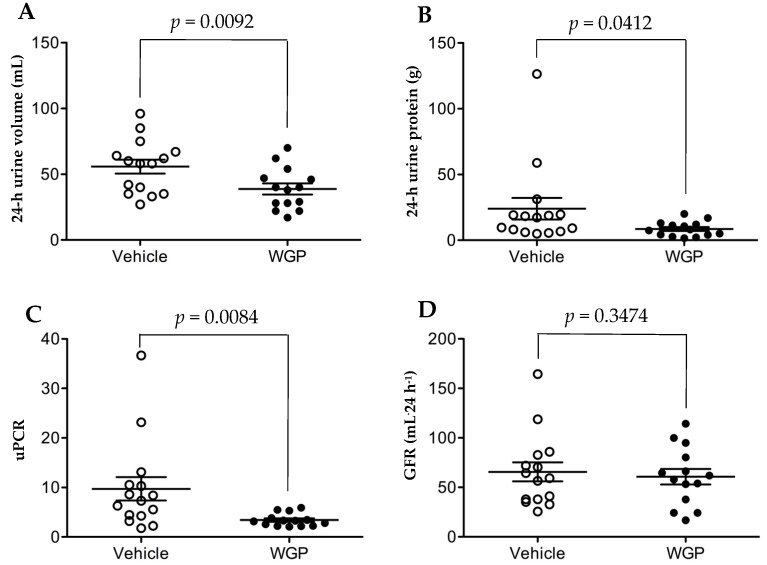
Daily intake of WGP reduces urine production and proteinuria but no effect on GFR in obese diabetic ZSF1 rats. A 24-h urine sample was collected from each rat in both Vehicle (*n* = 15) and WGP (*n* = 14) at the end of six months of dietary supplements. (**A**) Total volume of urine production from each rat during 24 h. Vehicle vs. WGP: *p* = 0.0092 (one-tailed *t*-test); (**B**) Total amount of protein in 24-h urine sample of each rat. Vehicle vs. WGP: *p* = 0.0412 (one-tailed *t*-test); (**C**) Protein to creatinine ratio (uPCR) in the urine sample of each rat. Vehicle vs. WGP: *p* = 0.0084 (one-tailed *t*-test); (**D**) GFR of each rat calculated based on creatinine clearance. Vehicle vs. WGP: *p* = 0.3474 (one-tailed *t*-test). Line: mean with the standard error of the mean (SEM).

The baseline ranges for renal function in obese ZSF1 rats at the age of eight weeks were 164.8 ± 14.5 mL·kg^−1^·day^−1^ of urine volume, 307 ± 23 mg·kg^−1^·day^−1^ of proteinuria or 5.98 ± 0.41 of urine protein to creatinine ratio (uPCR) [28], and the average of bodyweight of the rats at this age was approximately 300 g (Figure 1). After six months of feeding, a 24-h urine sample was collected from each rat. As shown in Figure 3A, WGP-fed rats had lower urine output than Vehicle controls (*p* = 0.0092, one-tailed *t*-test). Similarly, the WGP group had significantly lower proteinuria (*p* = 0.0412, one-tailed *t*-test) (Figure 3B), and uPCR (*p* = 0.0084, one-tailed *t*-test) (Figure 3C), when compared to the Vehicle controls. When GFR (mL·24 h^−1^) between these two groups was compared, no significant difference was found between the WGP-fed and control groups (*p* = 0.3474, one-tailed *t*-test) (Figure 3D). 

### 3.4. Daily Intake of WGP Partially Prevents Renal Pathological Changes in Obese Diabetic Rats 

**Figure 4:**
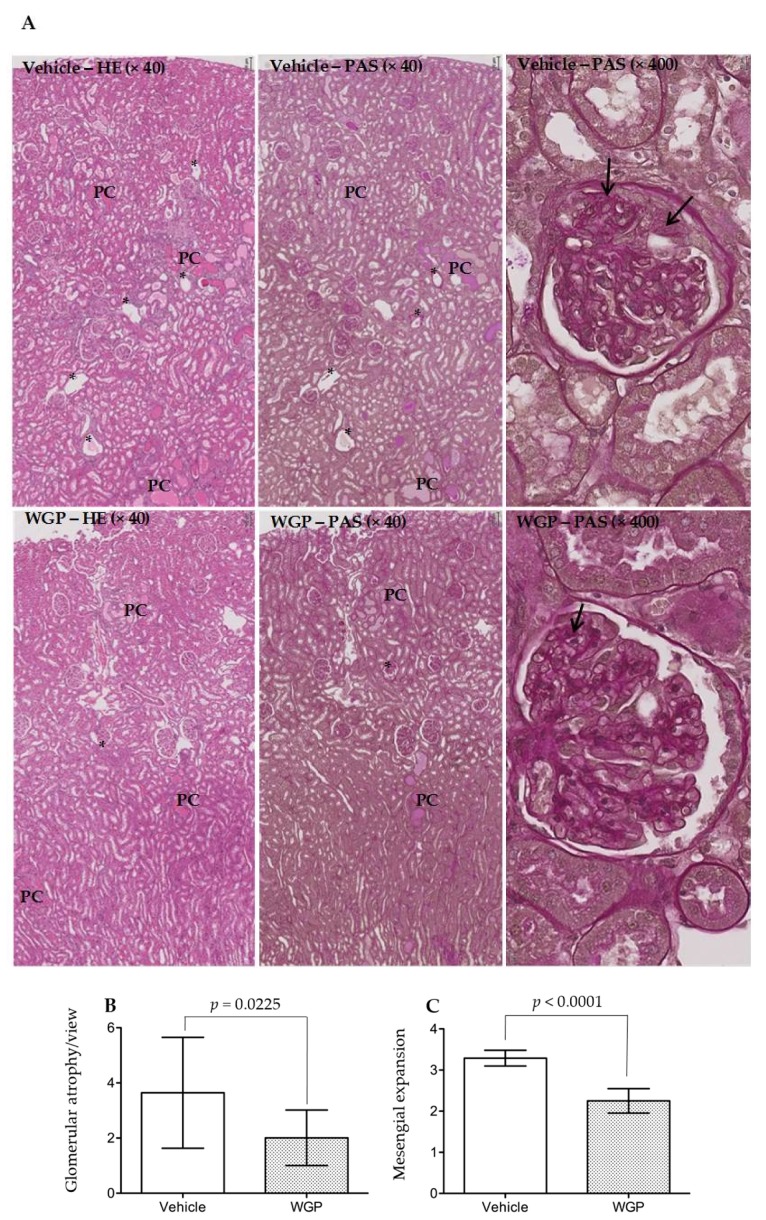

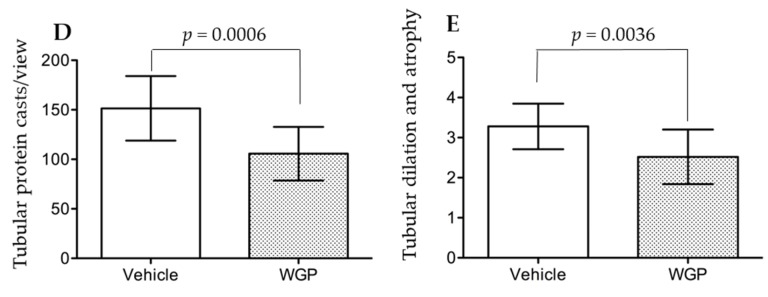
Daily intake of WGP reduces kidney injury in obese diabetic ZSF1 rats. At the end of six months of dietary supplements, six kidneys/rats were randomly selected from each group (Vehicle vs. WGP), and kidney sections were stained with either hematoxylin and eosin (HE) or Periodic acid-Schiff (PAS). (**A**) Typical microscopic images of renal cortex, outer medulla and a glomerulus in each group (Vehicle: top panel; WGP: bottom panel); data showed the same area of renal cortex and outer medulla stained with either HE or PAS. PC: protein cast formation; black stars (*): damaged glomerulus (glomerular atrophy); arrows: PAS-stained mesangial expansion; (**B**) The glomerular atrophy was scored in at least 20 randomly selected views in two separate sections of each kidney, and was presented in average per view; data are presented as mean ± SD of each group (*n* = 9); vehicle vs. WGP: *p* = 0.0225 (two-tailed *t*-test); (**C**) The mesangial expansion was determined using a 0 to 4 scale based on the percentage of the area stained strongly with PAS; a range of 180 to 250 glomeruli were counted and averaged for each kidney. Data are presented as mean ± SD of each group (*n* = 9); vehicle vs. WGP: *p* < 0.0001 (two-tailed *t*-test); (**D**) The number of intratubular protein cast formation in each microscopic view was counted in HE-stained section, and the average number of at least 20 randomly selected views under 40× magnification represented in each kidney; data are presented as mean ± SD of each group (*n* = 12); vehicle vs. WGP: *p* = 0.0006 (two-tailed *t*-test); (**E**) Tubular dilation and atrophy were determined using a 0–4 scale based on the percentage of damaged tubules occupying an area in each microscopic view; the average of at least 30 randomly selected views under 200× magnification represented the tubular atrophy in each kidney; data are presented as mean ± SD of each group (*n* = 12). Vehicle vs. WGP: *p* = 0.0036 (two-tailed *t*-test).

To further explore the beneficial effect of daily intake of WGP on the prevention of kidney damage in obese diabetic rats, histological analysis of kidney sections was performed and compared between these two groups. The histological score of all four renal pathological parameters in WGP group were significantly lower than that in Vehicle group (Figure 4A–E). The WGP group had lower scores of glomerular atrophy per view (*p* = 0.0225, two-tailed *t*-test, *n* = 9) (Figure 4B), reduced mesangial expansion (*p* < 0.0001, two-tailed *t*-test, *n* = 9) (Figure 4C), fewer tubular protein cast formation per view (*p* = 0.0006, two-tailed *t*-test, *n* = 12) (Figure 4D), and less severe tubular dilation and atrophy (*p* = 0.0036, two-tailed *t*-test, *n* = 12) (Figure 4E), when compared to the Vehicle controls. Together, the histological data indicated less kidney injury in all compartments of the kidneys in WGP-fed rats.

### 3.5. Daily Intake of WGP Is Associated with Increasing Local Antioxidant Defense in the Kidney of Obese Diabetic Rats 

To understand the mechanisms by which the daily intake of WGP reduced the progression of kidney disease in these rats, the transcriptional profile of 84 genes that are involved in oxidative stress and antioxidant defense in the kidney was examined using PCR array. In the kidneys of WGP-fed rats compared to Vehicle controls, the expression levels of antioxidants (*Gpx1, Gpx4, Gstk1 and Prdx2*) and reactive oxygen species (ROS) metabolism (*Sod2, Cyba, Dhcr24 and Park7*) were significantly up-regulated while oxidative stress response genes (*Hmox1, Ercc6, Gstp1 and Txnip*) downregulated (Table S2 and Table 2). These results suggest an increase in antioxidant defense in the kidneys by WGP diet. 

### 3.6. Addition of WGP Extract Reduced H_2_O_2_-Induced Cell Death in Cultured Podocytes 

**Figure 5:**
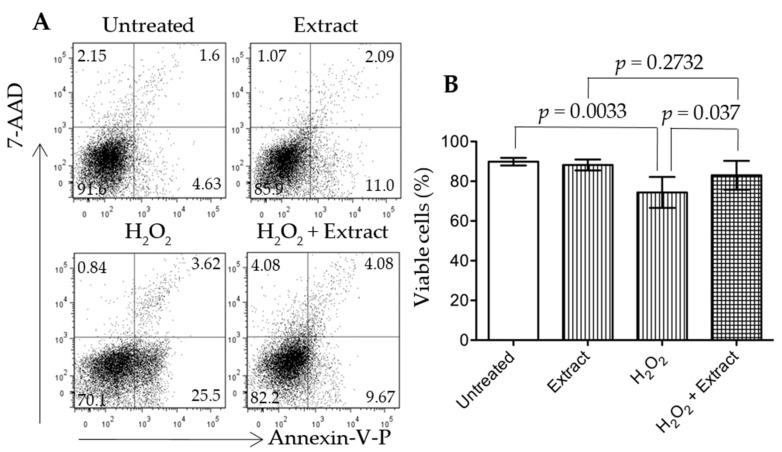
Grape extract protects cultured podocytes from H_2_O_2_-induced cell death. Grape extract was prepared by using methanol extraction. HSMP cells (0.25 × 10^6^ cells/well) were grown in RPMI 1640 culture medium in 24-well plates at 37 °C overnight, followed by grape extract treatment (200 mg·mL^−1^) in the presence or absence of H_2_O_2_ (1 μM). (**A**) Cell viability or apoptosis was determined by FACS analysis with Annexin-V-PE and 7-AAD staining after 24-h treatment with the grape extract. Data were represented as a typical FACS graph in each group; (**B**) Cell viability represented the percentage of viable cells (double-Annexin-V-PE/7-AAD negative cells in lower left quadrant). Data were presented as mean ± SD of eight separate experiments in each group. Untreated vs. H_2_O_2_, *p* = 0.0033 (two-tailed *t*-test); H_2_O_2_ vs. H_2_O_2_ + Extract, *p* = 0.037 (two-tailed *t*-test); or Extract vs. H_2_O_2_ + Extract, *p* = 0.2732 (two-tailed *t*-test).

Podocyte injury plays a key role in the initiation and early progression of DN [32]. To investigate if the antioxidant compounds from WGP prevented podocyte injury under oxidative stress, the protective activity of WGP extract was tested in cultured podocytes in the presence of H_2_O_2_. Methanol extraction of WGP exhibited a high antioxidative activity, indicated by the equivalence of approximately 1.7 mg of the extract to 5 μg of ascorbic acid in all three different assays (Figure S1). H_2_O_2_ induced significant podocyte cell death through apoptosis (positive stain with Annexin-V) (Figure 5A). However, cells that were co-treated with WGP extract (200 mg·mL^−1^) and H_2_O_2_ had significantly more cell viability than cells that were treated with H_2_O_2_ only (*p* = 0.037, two-tailed *t*-test, *n* = 8). The viability of cells treated with both H_2_O_2_ and WGP extract was not different from that in cultures treated with the extract only (*p* = 0.2732, two-tailed *t*-test. *n* = 8), suggesting that the extract significantly inhibited H_2_O_2_-induced cell death under this condition.

## 4. Discussion

CKD is a major health problem in our society. In addition to pharmacological therapy, such as through angiotensin converting enzyme (ACE) inhibitors or angiotensin receptor blockers (ARB) for proteinuria and/or hypertension control, nutritional interventions have been widely recommended in the management of CKD [33]. A low-protein diet is a popular and practical nutritional management of CKD [34,35,36]. Interestingly, a recent pilot clinical study shows that dietary supplementation with two gram of grape seed extract a day for six months can improve some kidney function parameters of 2–4 stage CKD in patients, in which the grape seed extract likely acts through the strong antioxidative activity and anti-inflammation of phytochemicals [37]. This study may suggest that a diet containing the antioxidative grape extract can potentially become a nutritional management strategy for the prevention of CKD. 

In the present study, when the rats started diet supplement at 8 weeks old, compared to the reference range [38] (Table S3), they had significant higher levels of blood GLU, BUN, TBIL, ALT, ALP and AMY, which may represent a sign of mild diabetes (higher GLU), pancreatic (higher AMY) and nonalcoholic fatty liver disease (higher ALT, ALP, TBIL and BUN). During six months of feeding experiments, the serum levels of AMY, Ca^2+^, GLOB, GLU, Na^+^, TBIL and TB were increased and of ALB (including ALB/GLOB ratio), ALP, BUN, K^+^ and PHOS decreased in both groups (Table S1). These data may suggest progressively worsening diabetic (high GLU) and pancreatic (high AMY), fatty liver (high GLOB, TB and TBIL, low ALB and BUN), and kidney disease (low ALB or ALB/GLOB ratio for albumin loss, and the imbalance of Na^+^, Ca^2+^, K^+^ and PHOS). When compared to the vehicle control group, with no difference in their bodyweight (Figure 1) the lower organ index of WGP-fed rats may indicate the reduced fatty liver, kidney hypertrophy and perhaps low-degree systemic immune response activity (indicated by the smaller size of the spleens) (Table 1). Further chemical tests showed that daily intake of WGP improved blood ALB and ALB/GLOB ratio and decreased GLU levels (Figure 2), and lower proteinuria, uPCR and urination (probably due to higher renal water reabsorption) (Figure 3). Histologically, we found that glomerular atrophy, mesangial expansion, tubular injury and protein cast formation, all of which are elevated in early stages of diabetic related CKD [39,40], were reduced in the WGP-fed rats (Figure 4). In vitro, the grape extract inhibited H_2_O_2_-induced cell death in cultured podocytes and exhibited a high antioxidative activity (Figure 5). In consistent with our study, a recently study demonstrates similar benefits of the grape powder to age-related kidney functions in Fischer 344 rats [21].

We described higher urine volumes in the vehicle fed rats versus WGP fed rats (Figure 3A), which may be due to either a manifestation of higher glycemic levels (Figure 2C) or worsened MetS, resulting in increasing hyperfiltration and renal injury. Hyperglycemia-induced hyperfiltration is an early manifestation of diabetes that causes polyuria and is an early predictor of glomerular damage in late stages of kidney disease [41,42,43]. While there was no evidence of better renal function (eGFR levels) in the WGP group (Figure 3D), this may have been due to the short duration of the study, since decline in GFR is a late marker of nephropathy when around 50% of nephrons are already lost [39,44]. The obese ZSF1 rats in this study indeed show the signs of early stages of CKD where an evidence of kidney damage such as proteinuria is present with normal or slightly decreased eGFR.

The renal protective pathway of WGP diet in obese diabetic ZSF1 rats is not fully understood. A previous study has demonstrated that the kidney disease of these rats is mostly independent on hypertension and/or hypertensive nephropathy [45], suggesting the attribution of renal pathophysiology strictly to other components of MetS, such as obesity and/or hyperglycemia. Indeed, many studies in literature use these rats as an appropriate model for the purposes of investigating MetS-related kidney disease or DN [28,46]. Our data show that the daily intake of WGP had no impact on the bodyweight or obesity of these rats (Figure 1). Therefore, the beneficial effect of WGP diet on the diseased kidney may act through direct and/or indirect pathways, which include the improvement of glucose metabolism and/or fatty liver disease (Figure 2), and the probability of suppressing low-degree immune response activity as indicated by the smaller size of the spleens (Table 1), but not relate to hypertension and obesity. The mechanism by which a WGP diet protects the kidney under the influence of both diabetic mellitus and fatty liver disease remains unknown and requires further investigation. 

Oxidative stress is induced by the excess levels of ROS that are generated in many different situations, including the mitochondrial injury [47], the increased activity of oxidases (e.g., cytochrome p450s, xanthine oxidase, 5-lipoxygenase, and NADPH oxidase) in the response to inflammatory stimuli (e.g*.*, hypoxia and immunologic stimuli) [48,49,50] and the loss of antioxidant defense system that includes enzymes (e.g., SOD, CAT and GSH-Px) and small molecular antioxidant scavengers (e.g., vitamin E, *N*-acetylcysteine and α-lipoic acid) [51,52]. The oxidative stress or ROS causes cellular damage by reacting with and/or denaturing cellular macromolecules including lipid, protein and nucleic acids, and/or even mediating or activating intracellular death signaling pathways [53], which plays an important role in the pathogenesis and progression of diabetes, fatty liver disease and their associated CKD including DN [52,54,55,56]. Grape products are rich in antioxidant chemicals, such as phenolic, flavonoid, and anthocyanidin [57,58,59], and exhibit anti-oxidative activities in both humans and animals [37,60,61]. Our study showed that each gram of WGP extract from methanol extraction contained approximately 21.5 mg of phenolics (reference: tannic acid), 0.25 mg of total flavonoids (reference: quercetin) and 5.2 mg of proanthocyandins (reference: catechine). The antioxidative activity of approximately 1.7 mg of this extract was equivalent to that of 5 μg of ascorbic acid in all three different assays (ABTS, DPPH, and FRAP) (Figure S1), and addition of this extract prevented H_2_O_2_-induced podocyte injury in cultures (Figure 5). Consist with these data, the anti-oxidative stress system in the kidneys of the rats receiving WGP diet was improved (Table 2). Taken together, these findings from both the literature and our study imply that WGP diet may improve antioxidant capability not only systemically (e.g., in the liver and the immune system/spleen and glucose metabolism) but also locally specific in the kidneys, resulting in less cellular damage in the setting of MetS and CKD.

The US Department of Agriculture considers 1.5 to 2 cups (250 g to 340 g, or 3.125 g·kg^−1^ to 4.25 g·kg^−1^ based on 80 kg, an average bodyweight of an adult) of fresh grapes per day as a standard serving size. In this experimental study, each ZSF1 rat (300 g to 600 g) consumed an average of approximately 25 g of food or 1.25 g of WGP a day that was equal to 2.8 g·kg^−1^·day^−1^. There are two simple ways to converse this dose between rats and humans [62]; one is based on the equivalent surface area (1 for rats = 1/7 for humans), so that the equivalent dose for humans will be 2.8 × 1/7 = 0.4 g·kg^−1^; the other is based on the representative surface area to weight ratio (Km), whereas rat Km = 5.9 and human (adult) Km = 37, then the dose for adult humans will be 2.8 × (5.9/37) = 0.45 g·kg^−1^. The result from both calculations is similar. Given that grapes are approximately 80% water by weight, 0.45 g·kg^−1^ of WGP dose equals to 2.25 g·kg^−1^ of fresh grapes, indicating that the dose we used in this experimental study was less than the standard serving size recommended by the US Department of Agriculture.

There are several limitations of this study. First, this is an experimental study using a relatively small number of animals from one strain, so the results may just limit to this strain and disease type. It is not certain if the same results would be seen in different species, or in different models of kidney disease. Second, a relatively short duration of this study may not represent the most of metabolic conditions and strategies that require life-long exposure and long duration of intervention. Finally, if the findings of the renoprotective effects of WGP diet are possibly attributable to better glycemic control, so there is a need to test the WGP diet in non-diabetic animal models. 

## 5. Conclusions

CKD has been known as an initially silent condition where clinical manifestations are usually underreported at its early stages. By the time SCr reaches abnormally high levels, there already is 50% nephron loss in the kidneys [44]. This highlights the importance of early stage management, such as lifestyle and dietary modifications, which may be able to improve disease prognosis [63]. In this experimental study, we demonstrate that a daily intake of WGP for six months shows renoprotection in a MetS animal model (obese diabetic ZSF1 rats), as evidenced by decreased proteinuria, lower uPCR, improved diabetic uremia and reduced renal injury in DN (early stages of CKD). Our findings suggest that a daily intake of grape product rich in antioxidants may delay the progression of early CKD to late or ESRD in humans, and warrants further investigation. 

## Figures and Tables

**Table 1:** Organ index (organ weight/bodyweight) after six months of feeding sugar (Vehicle) or whole grape powder (WGP).

	Kidneys	Liver	Spleen	Heart	Lung
Vehicle (*n* = 15)	0.0070 ± 0.00072	0.0627 ± 0.00418	0.00135± 0.00010	0.00289 ± 0.00115	0.00356 ± 0.00096
WGP (*n* = 14)	0.0063 ± 0.00069	0.0544 ± 0.00546	0.00129 ± 0.00001	0.00251 ± 0.00022	0.00371 ± 0.00103
*p* value *	0.0127	<0.0001	0.0341	0.2351	0.6880

* The difference between Vehicle and WGP was compared using two-tailed *t*-test.

**Table 2:** Significant changes of oxidative stress-related gene expression in renal cortex of WGP-fed rats as compared to Vehicle controls, analyzed using PCR array.

Gene Symbol	Gene Names	Functions	Fold Change *	*p* Value (*n* = 4)
*Dhcr24*	24-dehydrocholesterol reductase	H_2_O_2_ scavenger, preventing H_2_O_2_-induced cell death	4.265	0.00222
*Cyba*	Cytochrome *b*-245, alpha polypeptide	NADPH oxidase subunit, optimizing immunity	4.215	0.00875
*Gstk1*	Glutathione S-transferase kappa 1	Cellular detoxification (lipid peroxide detoxification)	1.1475	0.01279
*Prdx2*	Peroxiredoxin 2	H_2_O_2_ and Alkyl hydroperoxide antioxidant	1.7625	0.02746
*Sod2*	Superoxide dismutase, mitochondrial	Limiting ROS detrimental effect, and moderating ROS release	2.3375	0.02983
*Park7*	Parkinson disease (autosomal recessive, early onset) 7	Redox-sensitive chaperone	1.24	0.03723
*Gpx4*	Glutathione peroxidase 4	H_2_O_2_, lipid peroxide and hydroperoxide reduction	1.96	0.03756
*Gpx1*	Glutathione peroxidase 1	H_2_O_2_ antioxidant	3.235	0.04614
*Hmox1*	Heme oxygenase (decycling) 1	Heme degradation to CO	−101.533	0.01283
*Ercc6*	Excision repair cross-complementing rodent repair deficiency, complementation group 6	Damaged DNA repair	−3.9575	0.02908
*Gstp1*	Glutathione S-transferase pi 1	Cellular detoxification	−22.4875	0.0405
*Txnip*	Thioredoxin interacting protein	Increasing ROS production	−37.8825	0.04436

* Minus: decreased.

## Supplementary Materials

The following are available online at http://www.mdpi.com/2072-6643/9/4/345/s1, Figure S1: Antioxidant activity of WGP extract, Table S1: The blood chemistry of obese male ZSF1 rats over six months of daily intake of whole grape powder (WGP) compared to sugar (Vehicle) group, Table S2: Oxidative stress-related gene expression in kidney cortex of WGP-fed rats compared to Vehicle controls at the end of six months of feeding experiment, Table S3: Blood chemistry of metabolism of obese male ZSF1 rats (approximately eight weeks old) compared with a reference in the literature [36].
