# Supplementary material for: Daily Intake of Grape Powder Prevents the Progression of Kidney Disease in Obese Type 2 Diabetic ZSF1 Rats
Source: Nutrients. 2017 Mar 31;9(4):345. doi: 10.3390/nu9040345 (PMC5409684; doi:10.3390/nu9040345)
Supplement: Supplementary file 1 [file nutrients-09-00345-s001.docx]

**Table S1.** The blood chemistry of obese male ZSF1 rats over 6 months of daily intake of whole grape powder (WGP) compared to sugar (Vehicle) group.

| **Name and its initial levels (*n* = 4)** | **Months** | **Vehicle**  **(*n* = 7–12)** | **WGP**  **(*n* = 7–12)** | **WGP vs. Vehicle *** | **0–6 months in Vehicle **** | **0–6 months in WGP **** |
| --- | --- | --- | --- | --- | --- | --- |
| ALB (g·L^–1^):  55.5 ± 3.7 | 1 | 60.13 ± 2.42 | 60.75 ± 2.96 | 0.0013 | <0.0001 | <0.0001 |
|  | 3 | 52.67 ± 4.42 | 57.64 ± 3.72 |  |  |  |
|  | 6 | 46.09 ± 2.74 | 50.0 ± 4.27 |  |  |  |
| ALP (U·L^–1^):  617.25 ± 56.48 | 1 | 527.43 ± 103.15 | 478.14 ± 130.68 | 0.2075 | 0.0004 | <0.0001 |
|  | 3 | 389.75 ± 125.44 | 393.00 ± 72.70 |  |  |  |
|  | 6 | 347.91 ± 112.31 | 287.27 ± 76.61 |  |  |  |
| ALT (U·L^–1^):  89.75 ± 23.16 | 1 | 112.13 ± 26.77 | 85.88 ± 16.87 | 0.5368 | 0.8343 | 0.2369 |
|  | 3 | 104.6 ± 66.85 | 109.3 ± 33.75 |  |  |  |
|  | 6 | 117.0 ± 56.61 | 115.4 ± 43.35 |  |  |  |
| AMY (U·L^–1^):  904 ± 59.96 | 1 | 1035.14 ± 129.77 | 1017.71 ± 59.42 | 0.8423 | 0.0017 | <0.0001 |
|  | 3 | 1091.42 ± 117.43 | 1151.64 ± 79.22 |  |  |  |
|  | 6 | 1166.64 ± 90.0 | 1139.09 ± 71.78 |  |  |  |
| BUN(mmol·L^–1^):  8.08 ± 0.71 | 1 | 8.41 ± 0.52 | 8.09 ± 0.76 | 0.3378 | 0.0051 | 0.0005 |
|  | 3 | 6.65 ± 1.25 | 7.04 ± 0.41 |  |  |  |
|  | 6 | 7.45 ± 1.12 | 6.69 ± 0.90 |  |  |  |
| Ca^2+^ (mmol·L^–1^);  2.87 ± 0.05 | 1 | 2.91 ± 0.09 | 2.90 ± 0.04 | 0.1755 | <0.0001 | <0.0001 |
|  | 3 | 2.83 ± 0.06 | 2.85 ± 0.07 |  |  |  |
|  | 6 | 3.22 ± 0.23 | 3.07 ± 0.12 |  |  |  |
| CRE (µmol·L^–1^):  23.75 ± 8.3 | 1 | 26.13 ± 12.36 | 25.75 ± 10.59 | 0.4214 | 0.3068 | 0.2703 |
|  | 3 | 32 ± 9.89 | 29.42 ± 10.6 |  |  |  |
|  | 6 | 25.09 ± 8.72 | 22 ± 6.44 |  |  |  |
| GLOB (g·L^–1^):  15.75 ± 2.5 | 1 | 18.14 ± 3.67 | 16 ± 1.6 | 0.0521 | <0.0001 | <0.0001 |
|  | 3 | 22.75 ± 2.87 | 21.6 ± 1.52 |  |  |  |
|  | 6 | 32.67 ± 2.34 | 30.75 ± 2.71 |  |  |  |
| GLU (mmol·L^–1^):  9.3 ± 1.8 | 1 | 11.84 ± 3.81 | 10.05 ± 1.13 | 0.0276 | 0.0080 | 0.0039 |
|  | 3 | 17.1 ± 5.71 | 14.04 ± 3.95 |  |  |  |
|  | 6 | 18.89 ± 6.69 | 15.18 ± 4.08 |  |  |  |
| K^+^ (mmol·L^–1^):  6.93 ± 0.55 | 1 | 5.79 ± 0.7 | 5.74 ± 0.77 | 0.1415 | 0.0010 | 0.0437 |
|  | 3 | 3.93 ± 1.83 | 5.23 ± 1.12 |  |  |  |
|  | 6 | 5.23 ± 0.92 | 5.36 ± 1.16 |  |  |  |
| Na^+^ (mmol·L^–1^):  137.5 ± 4.04 | 1 | 138.63 ± 2.45 | 139.75 ± 1.67 | 0.0506 | <0.0001 | <0.0001 |
|  | 3 | 131.92 ± 3.29 | 134.91 ± 3.15 |  |  |  |
|  | 6 | 143.09 ± 3.48 | 143.64 ± 3.04 |  |  |  |
| PHOS (mmol·L^–1^):  3 ± 0.26 | 1 | 2.5 ± 0.17 | 2.45 ± 0.27 | 0.6585 | <0.0001 | 0.0029 |
|  | 3 | 1.46 ± 0.36 | 1.65 ± 0.21 |  |  |  |
|  | 6 | 2.10 ± 0.82 | 2.17 ± 1.03 |  |  |  |
| TBIL (µmol·L^–1^):  6 ± 0 | 1 | 11.5 ± 2.74 | 9.57 ± 2.99 | 0.4068 | 0.0101 | 0.0005 |
|  | 3 | 21.83 ± 8.31 | 21.64 ± 8.16 |  |  |  |
|  | 6 | 17.18 ± 10.97 | 13.91 ± 7.25 |  |  |  |
| TP (g·L^–1^):  70.75 ± 1.5 | 1 | 76.25 ± 3.66 | 77 ± 4.04 | 0.3532 | 0.0133 | 0.0013 |
|  | 3 | 76.67 ± 4.87 | 78.42 ± 2.91 |  |  |  |
|  | 6 | 78.91 ± 3.51 | 79.18 ± 3.66 |  |  |  |
| ALB/GLOB ratio:  3.64 ± 0.96 | 1 | 3.39 ± 0.68 | 3.82 ± 0.29 | 0.0092 | <0.0001 | <0.0001 |
|  | 3 | 2.26 ± 0.3 | 2.63 ± 0.11 |  |  |  |
|  | 6 | 1.44 ± 0.15 | 1.61 ± 0.23 |  |  |  |

ALB: albumin, ALP: alkaline phosphatase, ALT: alanine aminotransferase, AMY: amylase, BUN: blood urea nitrogen, CRE: creatinine, GLOB: globulin, GLU: glucose, PHOS: phosphate, TBIL: total bilirubin, TP: total protein. *Two-way ANOVA was used to analyze the difference of two groups at different time points (1–6 months), and **one-way ANOVA the change in each group over this time period (0–6 months).

**Table S2**. Oxidative stress-related gene expression in kidney cortex of WGP-fed rats compared to Vehicle controls at the end of 6 months of feeding experiment.

| **Gene** | **Fold Change** | ***p* value (*n* = 4)** | **Functional Gene Grouping** | | |
| --- | --- | --- | --- | --- | --- |
|  |  |  | **Antioxidant** | **ROS metabolism** | **O_2_ transporter** |
| *Alb* (Albumin) | -4.765 | 0.8290 | √ |  |  |
| *Als2*: (Amyotrophic lateral sclerosis 2 homolog) | -1.8525 | 0.3622 |  | √ |  |
| *Aox1* (Aldehydre oxidase 1) | -3.815 | 0.5276 |  | √ |  |
| *Apc* (Adenomatous polyposis coli) | -10.6175 | 0.1296 | √ |  |  |
| *Apoe* (Aolipoprotein E) | 1.0475 | 0.1594 |  | √ |  |
| *Cat* (Catalase) | - 2.64 | 0.6301 | √ | √ |  |
| *Ccl5* (Chemokine ligand 5, C-C motif) | -1.91 | 0.2797 |  | √ |  |
| *Ccs* (Copper chaperone for superoxide dismutase) | -0.13 | 0.0672 |  | √ |  |
| *Ctsb* (Cathepsin B) | -5.4925 | 0.917 | √ | √ |  |
| *Cyba* (Cytochrome b-245, alpha polypeptide) | 4.215 | 0.0088 |  | √ |  |
| *Cygb* (Cytoglobin) | -0.615 | 0.0523 |  |  | √ |
| *Dhcr24* (24-dehydrocholesterol reductase) | 4.265 | 0.0022 |  | √ |  |
| *Dnm2* (Dynamin 2) | -0.9575 | 0.2544 |  |  | √ |
| *Duox1* (Dual oxidase 1) | -10.5875 | 0.3824 | √ | √ |  |
| *Duox2* (Dual oxidase 2) | -9.005 | 0.6283 |  | √ |  |
| *Ehd2* (EH-domain containing 2) | -27.43 | 0.2511 |  |  |  |
| *Epx* (Eosinophil peroxidase) | -2.1675 | 0.1087 | √ | √ |  |
| *Ercc2* (Excision repair cross-complementing rodent repair deficiency, complementation group 2) | -34.055 | 0.7114 |  | √ |  |
| *Ercc6* (Excision repair cross-complementing rodent repair deficiency, complementation group 6) | -3.9575 | 0.0291 |  | √ |  |
| *Fancc* (Fanconi anemia, complementation group C) | -6.3375 | 0.7136 |  |  | √ |
| *Fmos* (Flavin containing monooxygenase 2) | -1.7375 | 0.2648 |  | √ |  |
| *Fth1* (Ferritin, heavy polypeptide 1) | -1.27 | 0.0632 |  | √ |  |
| *Gclc* (Glutamate-cysteine ligase, catalytic subunit) | -5.1575 | 0.8836 |  | √ |  |
| *Gclm* (Glutamate-cysteine ligase, modifier subunit) | -1.8975 | 0.2811 |  | √ |  |
| *Gpx1* (Glutathione peroxidase 1) | 3.235 | 0.0461 | √ | √ |  |
| *Gpx2* (Glutathione peroxidase 2) | 1.095 | 0.2012 | √ | √ |  |
| *Gpx3* (Glutathione peroxidase 3) | -3.55 | 0.8203 | √ | √ |  |
| *Gpx4* (Glutathione peroxidase 4) | 1.97 | 0.0376 | √ | √ |  |
| *Gpx5* (Glutathione peroxidase 5) | -22.125 | 0.0985 | √ | √ |  |
| *Gpx6* (Glutathione peroxidase 6) | -20.15 | 0.5856 | √ | √ |  |
| *Gpx7* (Glutathione peroxidase 7) | 0.4725 | 0.1704 | √ | √ |  |
| *Gsr* (Glutathione reductase) | 1.1625 | 0.117 | √ | √ |  |
| *Gstk1* (Glutathione S-transferase kappa 1 | 1.1475 | 0.0128 | √ |  |  |
| *Gstp1* (Glutathione S-transferase pi 1) | -22.4875 | 0.0405 | √ |  |  |
| *Hba1* (Hemoglobin alpha, adult chain 2) | -1.4375 | 0.3056 |  |  | √ |
| *Hmox1* (Heme oxygenase 1) | -101.533 | 0.0128 |  | √ |  |
| *Hspa1a* (Heat shock 70kD protein 1A) | -90.83 | 0.6362 |  | √ |  |
| *Idh1*: (Isocitrate dehydrogenase 1, soluble | -14.57 | 0.8696 |  | √ |  |
| *Ift172* (Intraflagellar transport 172 homolog) | -3.0375 | 0.6638 |  |  | √ |
| *Krt1* (Keratin 1) | -8.0875 | 0.3262 |  | √ |  |
| *Loc367198* (Similar to Serine/threonine-protein kinase ATR) | -8.5575 | 0.8574 |  |  | √ |
| *Lpo* (Lactoperoxidase) | -34.11 | 0.9131 | √ |  |  |
| *Mb* (Myoglobin) | -7.99 | 0.4573 |  |  | √ |
| *Mpo* (Myeloperoxidase) | -11.345 | 0.685 | √ | √ |  |
| *Ncf1* (Neutrophil cytosolic factor 1) | -13.3625 | 0.7721 |  | √ |  |
| *Ncf2* (Neutrophil cytosolic factor 2) | -1.3175 | 0.347 |  | √ |  |
| *Ngb* (Neuroglobin) | -1.02 | 0.3338 |  |  | √ |
| *Nos2* (Nitric oxide synthase 2, inducible) | -10.295 | 0.3791 |  | √ |  |
| *Nox4* (NADPH oxidase 4) | -1.3475 | 0.227 |  | √ |  |
| *Noxa1* (NADPH oxidase activator 1) | -22.4125 | 0.2524 |  | √ |  |
| *Noxo1* (NADPH oxidase organizer 1) | -60.28 | 0 |  | √ |  |
| *Nqo1* (NAD(P)H dehydrogenase, quinone 1) | -14.7675 | 0.4142 |  | √ |  |
| *Nudt1* (Nudix -type) | 1.3625 | 0.1017 |  | √ |  |
| *Park7* (Parkinson disease 7) | 1.24 | 0.0372 |  | √ |  |
| *Prdx1* (Peroxiredoxin 1) | 0.8075 | 0.0671 | √ | √ |  |
| *Prdx2* (Peroxiredoxin 2) | 1.7625 | 0.0275 | √ | √ |  |
| *Prdx3* (Peroxiredoxin 3) | 0.0375 | 0.1921 | √ | √ |  |
| *Prdx4* (Peroxiredoxin 4) | -0.6625 | 0.0665 | √ | √ |  |
| *Prdx5* (Peroxiredoxin 5) | -2.4225 | 0.4794 | √ | √ |  |
| *Prdx6* (Peroxiredoxin 6) | -1.38 | 0.1691 | √ | √ |  |
| *Prnp* (Prion protein) | -2.2825 | 0.6121 |  | √ |  |
| *Psmb5*(Proteasome subunit, beta type 5) | 0.165 | 0.0531 |  | √ |  |
| *Ptgs1* (Prostaglandin-endoperoxide synthase 1) | -14.5025 | 0.6374 | √ |  |  |
| *Ptgs2* (Prostaglandin-endoperoxide synthase 2) | -9.6775 | 0.2504 | √ |  |  |
| *Rag2* (Recombination activating gene 2) | -65.135 | 0.5293 | √ |  |  |
| *Scd1* (Stearoyl-Coenzyme A desaturase 1) | -178.643 | 0.2158 |  | √ |  |
| *Vimp* (Selenoprotein S) | 1.6175 | 0.0703 | √ | √ |  |
| *Sepp1* (Selenoprotein P, plasma, 1) | -8.485 | 0.9872 |  | √ |  |
| *Serpinb1b* (Serine/cysteine peptidase inhibitor, clade B) | 0.655 | 0.1419 | √ |  |  |
| *Slc38a1*(Solute carrier family 38, member 1) | -22.135 | 0.9178 |  |  | √ |
| *Slc38a2* (Solute carrier family 38, member 5) | -20.5175 | 0.9212 |  |  | √ |
| *Sod1* (Superoxide dismutase 1, soluble) | -0.2225 | 0.0814 | √ | √ |  |
| *Sod2* (Superoxide dismutase 2, mitochondrial) | 2.3375 | 0.0298 |  | √ |  |
| *Sod3* (Superoxide dismutase 3, extracellular) | -0.9 | 0.1849 | √ | √ |  |
| *Sqstm1* (Sequestosome 1) | -0.665 | 0.1663 |  | √ |  |
| *Srxn1* (Sulfiredoxin 1 homolog | -4.545 | 0.0856 | √ |  |  |
| *Tpo* (Thyroid peroxidase) | -4.92 | 0.5533 |  | √ |  |
| *Txn1* (Thioredoxin 1) | -1.2175 | 0.0805 |  | √ |  |
| *Txnip* (Thioredoxin interacting protein) | -37.8825 | 0.0444 |  | √ |  |
| *Txnrd1* (Thioredoxin reductase 1) | -8.78 | 0.4777 | √ | √ |  |
| *Txnrd2* (Thioredoxin reductase 2) | -12.6275 | 0.8172 | √ | √ |  |
| *Ucp2* (Uncoupling protein 2) | -1.5775 | 0.0723 |  | √ |  |
| *Ucp3*(Uncoupling protein 3) | -10.865 | 0.8321 |  | √ |  |
| *Vim* (Vimentin) | 1.875 | 0.0703 |  |  | √ |

The levels of oxidative stress-related genes were analyzed using rat oxidative stress PCR array (Catalog No. PAMM-120Z, QIAGEN, Toronto, ON, Canada). Positive in fold change: up-regulated; negative in fold change: down-regulated; “√”: member of a functional group.

**Table S3.** Blood chemistry of metabolism of obese male ZSF1 rats (approximately 8 weeks old) compared with a reference in literature [36].

| **Blood chemistry** | **Obese male ZSF1 rats ( *n* = 4)** | **Reference *** |
| --- | --- | --- |
| Total protein (TP, g·L^–1^) | 70.75 ± 1.5 | 61 ± 2.1 |
| Albumin (ALB, g·L^–1^) | 55.5 ± 3.7 | 35.7 ± 0.84 |
| Glucose (GLU, mmol·L^–1^) | 9.3 ± 1.8 | 4.97 ± 1.4 |
| Blood urea nitrogen (BUN, mmol·L^–1^) | 8.08 ± 0.71 | 5.78 ± 0.98 |
| Creatinine (Cre, μmol·L^–1^) | 23.75 ± 8.3 | 35.18 ± 2.73 |
| Sodium (Na^+^, mmol·L^–1^) | 137.5 ± 4.04 | 144.9 ± 1.97 |
| Potassium (K^+^, mmol·L^–1^) | 6.93 ± 0.55 | 4.5 ± 0.2 |
| Phosphorous (PHOS, mmol·L^–1^) | 3 ± 0.26 | 4.55 ± 0.42 |
| Calcium (Ca^2+^, mmol·L^–1^) | 2.87 ± 0.05 | 2.69 ± 0.07 |
| Total bilirubin (TBIL, mg·L^–1^) | 6 ± 0 | 3.4 ± 0.36 |
| Alanine aminotransferase (ALT, U·L^–1^) | 89.75 ± 23.16 | 37 ± 6.4 |
| Alkaline phosphatase (ALP, U·L^–1^) | 617.25 ± 56.48 | 628 ± 123.4 |
| Amylase (AMY , U·L^–1^) | 904 ± 59.96 | 3427 ± 696.6 |
| Globulin (GLOB, g·L^–1^) | 15.75 ± 2.5 | 25 ± 1.5 |
| ALB/GLOB ratio | 3.64 ± 0.96 | <1.5 |

* The blood samples were collected from the sublingual vein of adult male Sprague-Dawley rats (average bodyweight: 292 g; 8.5–11 weeks old, *n* = 40).


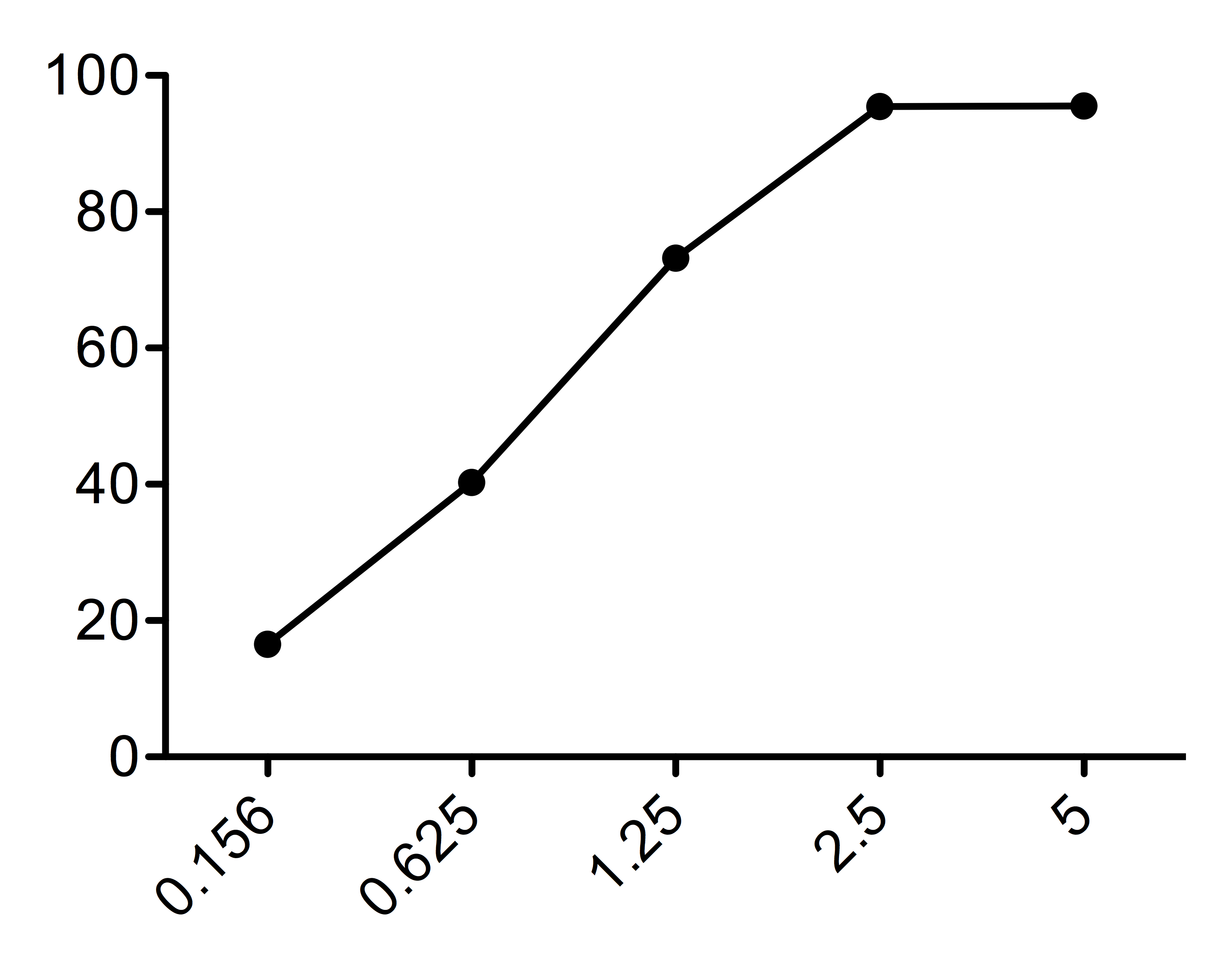

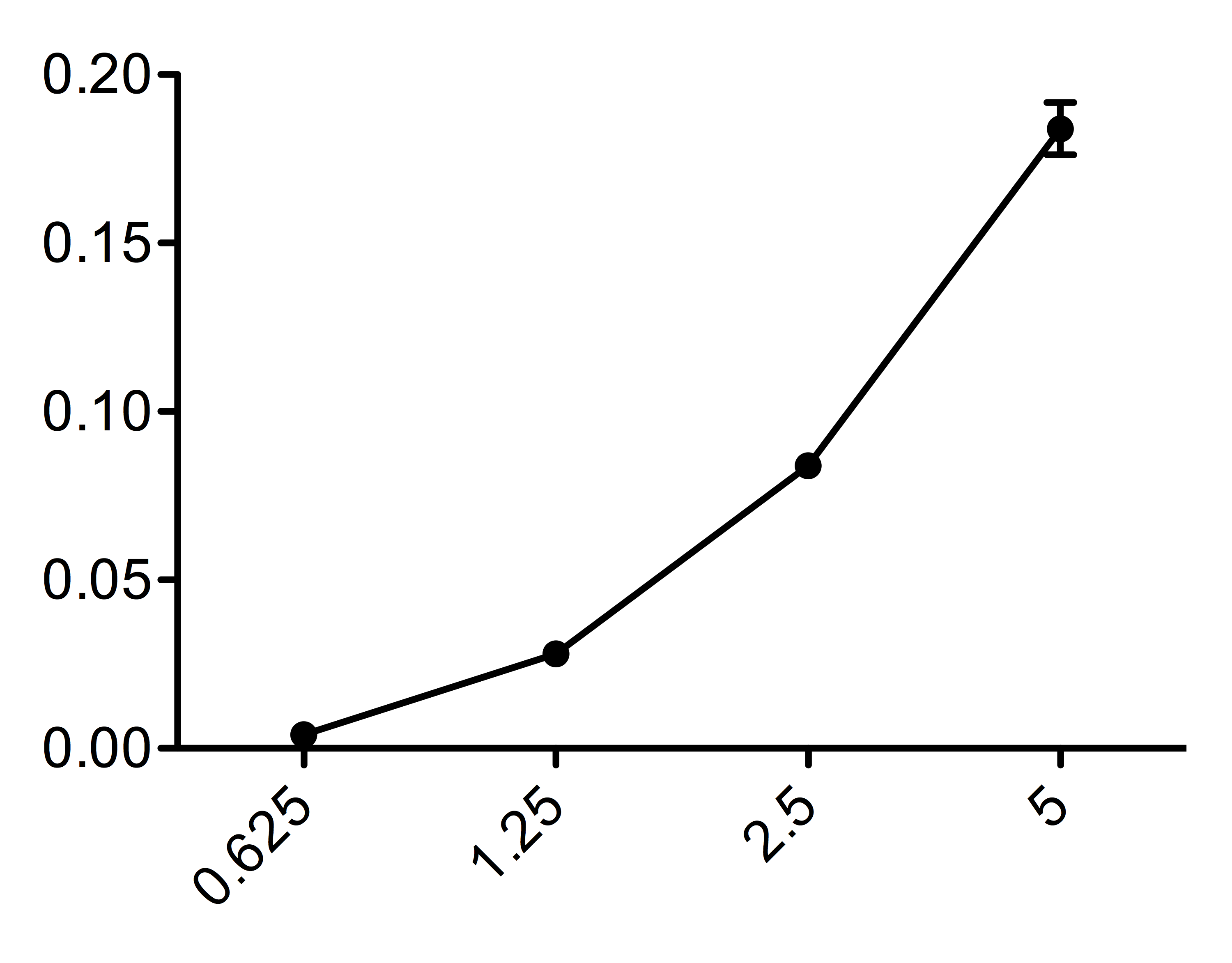


**B**

**A**

**Fe^2+^(mmol^·^L^-1^)**

**DPPH radical scavenging (%)**

**Grape extract (mg)**

**Grape extract (mg)**

**C**


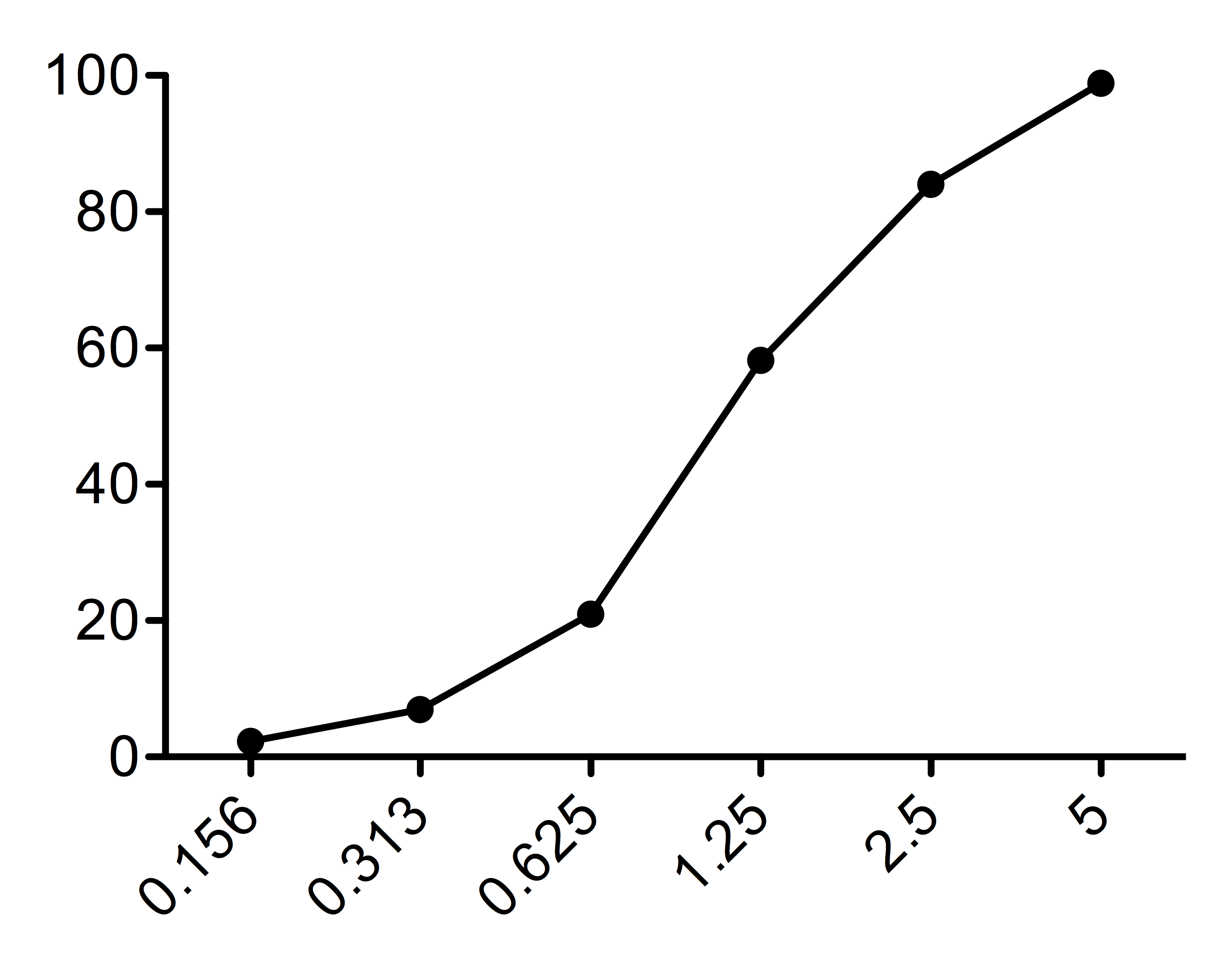


**ABTS scavenging (%)**

**Grape extract (mg)**

**Figure S1.** Antioxidant activity of WGP extract. WGP extract was prepared by methanol extraction, and its antioxidant activity was determined by using three different standard methods followed their standard protocols. (**A**) Ferric reducing ability of plasma (FRAP) assay. This assay was performed based on the ability of the extract to reduce ferric, so that it measured the antioxidants as ferric reductants where ferric was transformed to ferrous at low pH caused the formation of blue-colored ferrous-tripyridyltriazine complex. Data were presented as mean ± SD of three separate experiments. Five microgram of ascorbic acid was gave 0.047 ± 0.003 of Fe^2+^ (mmol·L^–1^) (equivalent to 1.7–1.8 mg of the extract) in this assy. (**B**) α, α-diphenyl-β-picrylhydrazyl (DPPH) free radical scavenging assay. DPPH assay measured radical scavenging activity of an antioxidant as DPPH radical accepts hydrogen. The diminishing of DPPH radical in test samples was indicated by the color change from purple to yellow. Data were presented as mean ± SD of three separate experiments. Five microgram of ascorbic acid was gave 89.07 ± 0.002% of DPPH radical scavenging (equivalent to 2 mg of the extract) in this assay. (**C**) 2,2'-azino-bis(3-ethylbenzothiazoline-6-sulphonic acid) (ABTS) scavenging assay. This assay was based the oxidation of ABTS (green) by H_2_O_2_ that was suppressed by antioxidants through electron donation radical scavenging, resulting in decreasing the green colored ABTS radical formation. Data were presented as mean ± SD of three separate experiments. Five microgram of ascorbic acid was gave 54.06% ± 0.031% of ABTS scavenging (equivalent to 1.2 mg of the extract) in this assay.
